# Supplementary material for: Short-Term Exposure to Nitrogen Dioxide Modifies Genetic Predisposition in Blood Lipid and Fasting Plasma Glucose: A Pedigree-Based Study
Source: Biology (Basel). 2023 Nov 27;12(12):1470. doi: 10.3390/biology12121470 (PMC10740487; doi:10.3390/biology12121470)
Supplement: Supplementary file 1 [file biology-12-01470-s001.zip › biology-2678865-supplementary.pdf]

Supplemental Material

Tables

Table S1. Percentage changes with 95% confidence intervals (95% CIs) in TG, TC, LDLC, HDLC and FPG associated with per 1 μg/m^3 increase in NO_2 of different lag structures among population stratified by sex.

Trait	Lag structure	Sex	Percentage changes (95%CI)	P	P for interaction
TG	Lag0	Male	0.072(-0.951,1.106)	0.890	0.865
		Female	0.050(-0.828,0.936)	0.911	
	Lag1	Male	0.325(-0.815,1.478)	0.578	0.185
		Female	0.964(-0.022,1.959)	0.056	
	Lag2	Male	0.461(-0.796,1.735)	0.474	0.445
		Female	1.081(-0.030,2.204)	0.057	
	Mv01	Male	0.286(-1.163,1.756)	0.701	0.402
		Female	0.177(-1.059,1.429)	0.780	
	Mv02	Male	1.215(-0.863,3.337)	0.254	0.428
		Female	0.125(-1.606,1.886)	0.889	
TC	Lag0	Male	0.143(-0.114,0.401)	0.276	0.556
		Female	0.275(-0.182,0.734)	0.239	
	Lag1	Male	0.019(-0.250,0.288)	0.892	0.368
		Female	0.318(-0.179,0.817)	0.210	
	Lag2	Male	0.035(-0.273,0.344)	0.822	0.203
		Female	0.455(-0.114,1.027)	0.118	
	Mv01	Male	0.147(-0.215,0.510)	0.427	0.488
		Female	0.222(-0.417,0.865)	0.496	
	Mv02	Male	0.188(-0.333,0.711)	0.481	0.363
		Female	0.385(-0.528,1.306)	0.411	
LDLC	Lag0	Male	0.229(-0.074,0.533)	0.139	0.828
		Female	0.243(-0.014,0.501)	0.064	
	Lag1	Male	0.079(-0.243,0.403)	0.630	0.573
		Female	0.226(-0.043,0.496)	0.100	
	Lag2	Male	0.251(-0.113,0.617)	0.177	0.728
		Female	0.194(-0.119,0.509)	0.226	
	Mv01	Male	0.198(-0.230,0.627)	0.366	0.626
		Female	0.081(-0.275,0.438)	0.656	
	Mv02	Male	0.014(-0.605,0.636)	0.966	0.638
		Female	0.036(-0.474,0.549)	0.890	
HDLC	Lag0	Male	-2.166(-2.851,-1.475)	<0.001	0.122
		Female	-1.690(-2.122,-1.257)	<0.001	
	Lag1	Male	-0.608(-1.294,0.083)	0.085	0.012

		Female	-1.083(-1.540,-0.623)	<0.001	
	Lag2	Male	-0.602(-1.332,0.134)	0.109	0.226
		Female	-0.412(-0.873,0.052)	0.082	
	Mv01	Male	-2.046(-2.945,-1.139)	<0.001	0.033
		Female	-1.881(-2.445,-1.314)	<0.001	
	Mv02	Male	-2.109(-3.395,-0.805)	0.002	0.056
		Female	-1.318(-2.092,-0.538)	<0.001	
GLU	Lag0	Male	-0.298(-0.621,0.026)	0.072	0.145
		Female	-0.054(-0.266,0.158)	0.618	
	Lag1	Male	-0.092(-0.417,0.235)	0.582	0.296
		Female	0.236(0.021,0.452)	0.032	
	Lag2	Male	0.080(-0.298,0.460)	0.679	0.813
		Female	0.116(-0.147,0.380)	0.387	
	Mv01	Male	-0.408(-0.856,0.042)	0.076	0.216
		Female	0.141(-0.150,0.432)	0.344	
	Mv02	Male	-0.515(-1.153,0.127)	0.116	0.458
		Female	0.196(-0.222,0.616)	0.358	

HDLC, high-density lipoprotein cholesterol; Lag0, concentrations of the day of investigation; Lag1, concentrations of the previous day of investigation; Lag2, concentra-tions of the two days before the investigation; LDLC, low-density lipoprotein cholesterol, Mv01, moving average concentrations of Lag0 and Lag1; Mv02, moving average concentrations of Lag0 to Lag2; TC, total cholesterol; TG, triglycerides.

Multivariable model was adjusted for age, sex, BMI, education level, family ID as random effect term, physical activity, smoking, drinking, vegetable and fruit intake, the whole grain intake, meat intake, tea intake, use of antihypertensive/antidiabetic/antihyperlipidemic drug, and concentration of $PM_{2.5}$, PM_{10}, CO and O_3.

Table S2. Percentage changes with 95% confidence intervals (95% CIs) in TG, TC, LDLC, HDLC and FPG associated with per 1 μg/m^3 increase in NO_2 of different lag structures among population stratified by age group.

Trait	Lag structure	Age	Percentage changes (95%CI)	P	P for interaction
TG	Lag0	<60	0.194(-0.725,1.123)	0.680	0.112
		>=60	0.182(-0.774,1.146)	0.711	
	Lag1	<60	0.054(-0.902,1.019)	0.913	0.028
		>=60	1.578(0.418,2.751)	0.008	
	Lag2	<60	0.427(-0.700,1.566)	0.460	0.079
		>=60	1.391(0.133,2.665)	0.030	
	Mv01	<60	0.554(-0.670,1.794)	0.377	0.052
		>=60	0.034(-1.419,1.508)	0.964	
	Mv02	<60	1.744(-0.075,3.597)	0.061	0.073
		>=60	0.174(-1.856,2.247)	0.868	
TC	Lag0	<60	0.086(-0.156,0.330)	0.486	0.527
		>=60	0.346(-0.190,0.885)	0.207	
	Lag1	<60	0.164(-0.084,0.413)	0.194	0.718
		>=60	0.249(-0.347,0.848)	0.414	
	Lag2	<60	0.038(-0.256,0.333)	0.799	0.629
		>=60	0.333(-0.337,1.008)	0.331	
	Mv01	<60	0.017(-0.304,0.339)	0.917	0.566
		>=60	0.297(-0.502,1.102)	0.468	
	Mv02	<60	0.348(-0.119,0.816)	0.145	0.526
		>=60	0.156(-0.967,1.292)	0.786	
LDLC	Lag0	<60	0.388(0.110,0.668)	0.006	0.727
		>=60	0.125(-0.153,0.404)	0.377	
	Lag1	<60	0.304(0.020,0.588)	0.036	0.754
		>=60	0.039(-0.266,0.345)	0.802	
	Lag2	<60	0.281(-0.054,0.616)	0.100	0.900
		>=60	0.121(-0.225,0.467)	0.494	
	Mv01	<60	0.095(-0.274,0.466)	0.614	0.698
		>=60	0.180(-0.230,0.592)	0.390	
	Mv02	<60	0.026(-0.516,0.572)	0.924	0.786
		>=60	0.118(-0.463,0.702)	0.691	
HDLC	Lag0	<60	-1.835(-2.293,-1.375)	<0.001	0.581
		>=60	-2.073(-2.712,-1.429)	<0.001	
	Lag1	<60	-1.286(-1.781,-0.788)	<0.001	0.261
		>=60	-0.426(-1.072,0.223)	0.198	
	Lag2	<60	-0.478(-0.959,0.006)	0.053	0.614
		>=60	-0.644(-1.331,0.048)	0.068	
	Mv01	<60	-2.158(-2.760,-1.553)	<0.001	0.418
		>=60	-1.772(-2.616,-0.920)	<0.001	
	Mv02	<60	-1.561(-2.392,-0.723)	<0.001	0.363
		>=60	-1.742(-2.921,-0.548)	0.004	

GLU	Lag0	<60	-0.247(-0.486,-0.007)	0.044	0.533
		>=60	-0.113(-0.406,0.180)	0.448	
	Lag1	<60	-0.034(-0.267,0.199)	0.776	0.866
		>=60	0.285(-0.019,0.591)	0.067	
	Lag2	<60	0.169(-0.120,0.459)	0.252	0.688
		>=60	-0.033(-0.386,0.321)	0.854	
	Mv01	<60	-0.297(-0.616,0.022)	0.068	0.665
		>=60	0.140(-0.279,0.560)	0.513	
	Mv02	<60	-0.378(-0.844,0.089)	0.113	0.640
		>=60	0.144(-0.441,0.733)	0.630	

HDLC, high-density lipoprotein cholesterol; Lag0, concentrations of the day of investigation; Lag1, concentrations of the previous day of investigation; Lag2, concentra-tions of the two days before the investigation; LDLC, low-density lipoprotein cholesterol, Mv01, moving average concentrations of Lag0 and Lag1; Mv02, moving average concentrations of Lag0 to Lag2; TC, total cholesterol; TG, triglycerides.

Multivariable model was adjusted for age, sex, BMI, education level, family ID as random effect term, physical activity, smoking, drinking, vegetable and fruit intake, the whole grain intake, meat intake, tea intake, use of antihypertensive/antidiabetic/antihyperlipidemic drug, and concentration of $PM_{2.5}$, PM_{10}, CO and O_3.

Table S3. Percentage changes with 95% confidence intervals (95% CIs) in TG, TC, LDLC, HDLC and FPG associated with per 1 μg/m^3 increase in NO_2 of different lag structures among population stratified by BMI.

Trait	Lag structure	BMI	Percentage changes (95%CI)	P	P for interaction
TG	Lag0	<24	0.783(-0.163,1.737)	0.105	0.957
		>=24	0.616(-0.373,1.614)	0.224	
	Lag1	<24	1.754(0.475,3.048)	0.007	0.147
		>=24	0.145(-0.827,1.126)	0.771	
	Lag2	<24	1.999(0.787,3.226)	0.001	0.770
		>=24	0.079(-1.124,1.297)	0.898	
	Mv01	<24	0.829(-0.620,2.299)	0.264	0.476
		>=24	0.024(-1.271,1.337)	0.971	
	Mv02	<24	0.255(-1.769,2.319)	0.807	0.665
		>=24	1.591(-0.286,3.504)	0.098	
TC	Lag0	<24	0.273(-0.180,0.727)	0.238	0.507
		>=24	0.279(0.024,0.536)	0.033	
	Lag1	<24	0.310(-0.175,0.798)	0.211	0.587
		>=24	0.005(-0.262,0.272)	0.972	
	Lag2	<24	0.384(-0.162,0.934)	0.169	0.636
		>=24	0.030(-0.283,0.344)	0.850	
	Mv01	<24	0.160(-0.479,0.803)	0.624	0.592
		>=24	0.271(-0.079,0.623)	0.129	
	Mv02	<24	0.239(-0.661,1.146)	0.604	0.607
		>=24	0.493(-0.022,1.010)	0.061	
LDLC	Lag0	<24	0.202(-0.058,0.463)	0.129	0.756
		>=24	0.248(-0.056,0.553)	0.111	
	Lag1	<24	0.211(-0.067,0.490)	0.138	0.815
		>=24	0.130(-0.187,0.447)	0.422	
	Lag2	<24	0.120(-0.196,0.436)	0.458	0.359
		>=24	0.363(-0.002,0.731)	0.052	
	Mv01	<24	0.076(-0.294,0.448)	0.687	0.722
		>=24	0.181(-0.234,0.599)	0.394	
	Mv02	<24	0.028(-0.492,0.551)	0.917	0.533
		>=24	0.009(-0.606,0.627)	0.978	
HDLC	Lag0	<24	-2.216(-2.755,-1.674)	<0.001	0.558
		>=24	-1.153(-1.637,-0.667)	<0.001	
	Lag1	<24	-1.118(-1.686,-0.547)	<0.001	0.766
		>=24	-0.414(-0.904,0.079)	0.100	
	Lag2	<24	-0.715(-1.278,-0.150)	0.013	0.706
		>=24	0.017(-0.520,0.557)	0.951	
	Mv01	<24	-2.253(-2.970,-1.532)	<0.001	0.645
		>=24	-1.162(-1.788,-0.532)	<0.001	
	Mv02	<24	-2.103(-3.094,-1.101)	<0.001	0.924
		>=24	-0.406(-1.303,0.500)	0.379	

GLU	Lag0	<24	-0.212(-0.441,0.017)	0.070	0.828
		>=24	-0.125(-0.439,0.191)	0.439	
	Lag1	<24	0.097(-0.138,0.333)	0.420	0.597
		>=24	0.021(-0.292,0.334)	0.896	
	Lag2	<24	0.048(-0.224,0.322)	0.729	0.515
		>=24	0.143(-0.240,0.528)	0.464	
	Mv01	<24	-0.146(-0.469,0.178)	0.377	0.779
		>=24	-0.130(-0.553,0.293)	0.546	
	Mv02	<24	-0.247(-0.698,0.207)	0.286	0.708
		>=24	-0.093(-0.712,0.529)	0.768	

HDLC, high-density lipoprotein cholesterol; Lag0, concentrations of the day of investigation; Lag1, concentrations of the previous day of investigation; Lag2, concentra-tions of the two days before the investigation; LDLC, low-density lipoprotein cholesterol, Mv01, moving average concentrations of Lag0 and Lag1; Mv02, moving average concentrations of Lag0 to Lag2; TC, total cholesterol; TG, triglycerides.

Multivariable model was adjusted for age, sex, BMI, education level, family ID as random effect term, physical activity, smoking, drinking, vegetable and fruit intake, the whole grain intake, meat intake, tea intake, use of antihypertensive/antidiabetic/antihyperlipidemic drug, and concentration of $PM_{2.5}$, PM_{10}, CO and O_3.

Figures

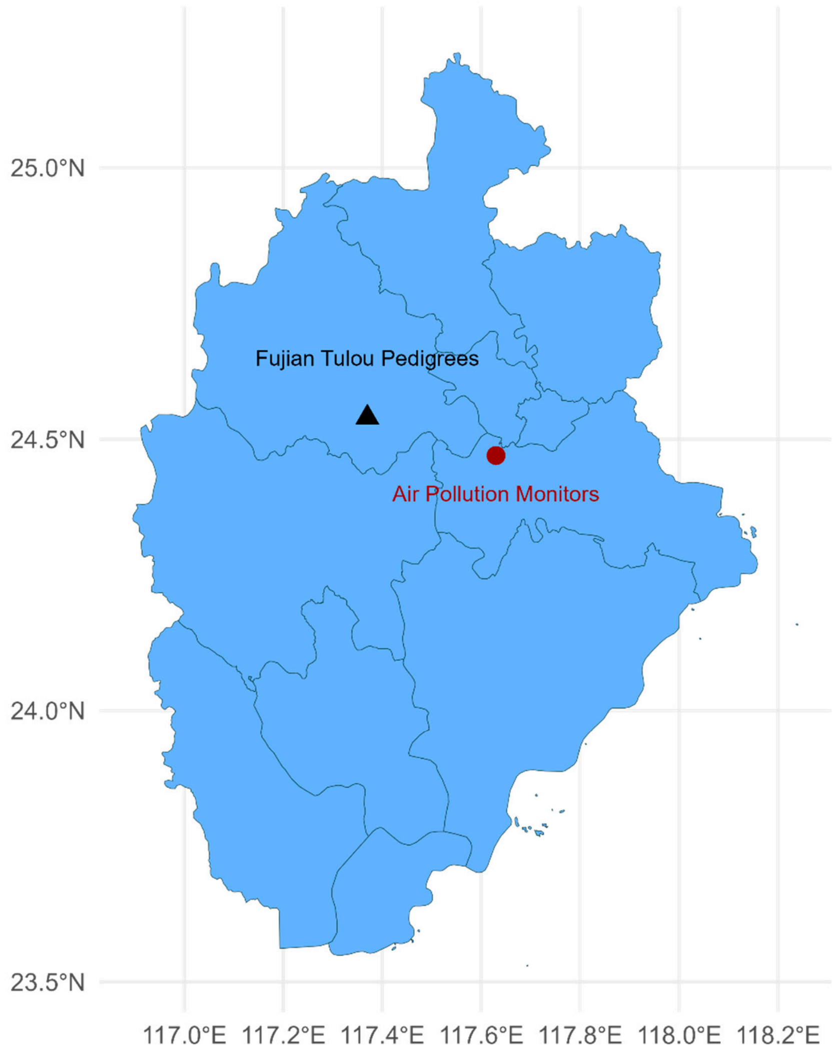

Figure S1. Map of Fujian Tulou Pedigree-based Cohort study site and air pollutant monitor locations. The black triangle represented the Fujian Pedigree-based Cohort site at 117.37°E, 24.54°N; the red dot represented the air pollutant monitors at 117.63°E, 24.47°N.

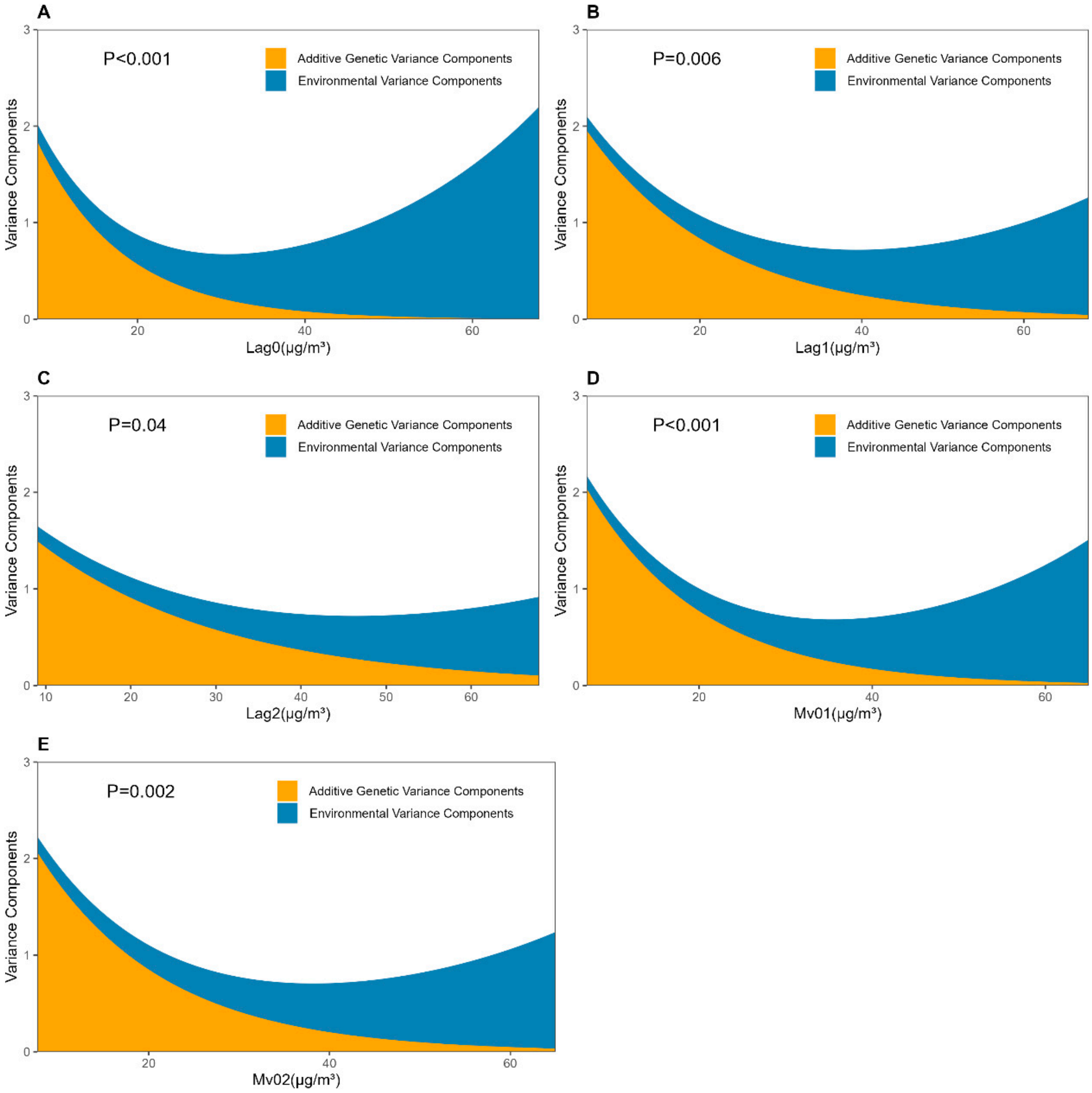

Figure S2. Interactions of genotype by NO_2 on HDLC in different lag structures. In all lag structures, environmental effects on HDLC increased with increasing NO_2 concentrations, while genetic effects diminished gradually. Lag0, concentrations of the day of investigation; Lag1, concentrations of the previous day of investigation; Lag2, concentrations of the two days before the investigation; Mv01, moving average concentrations of Lag0 and Lag1; Mv02, moving average concentrations of Lag0 to Lag2.

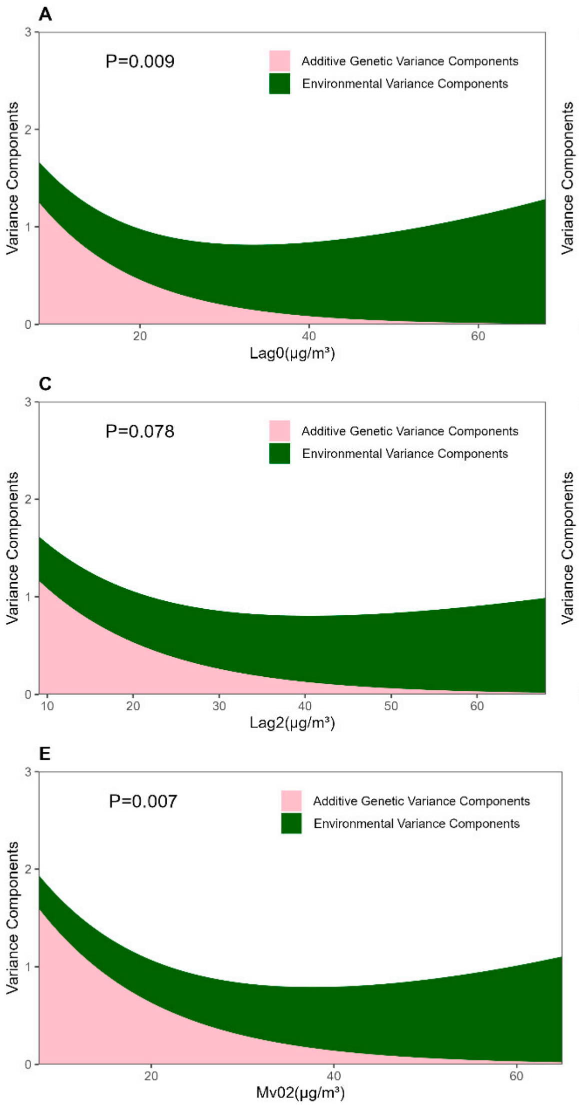

Figure S3. Interactions of genotype by NO_2 on FPG in different lag structures. In all lag structures, environmental effects on FPG increased with increasing NO_2 concentrations, while genetic effects diminished gradually. Lag0, concentrations of the day of investigation; Lag1, concentrations of the previous day of investigation; Lag2, concentrations of the two days before the investigation; Mv01, moving average concentrations of Lag0 and Lag1; Mv02, moving average concentrations of Lag0 to Lag2.
